# Effect of Ketamine on Postoperative Neurocognitive Disorders: A Systematic Review and Meta-Analysis

**DOI:** 10.3390/jcm12134314

**Published:** 2023-06-27

**Authors:** Dmitriy Viderman, Mina Aubakirova, Fatima Nabidollayeva, Nurgul Yegembayeva, Federico Bilotta, Rafael Badenes, Yerkin Abdildin

**Affiliations:** 1 Department of Biomedical Sciences, Nazarbayev University School of Medicine (NUSOM), Kerei and Zhanibek Khandar Str. 5/1, Astana 010000, Kazakhstan; drviderman@gmail.com (D.V.); mina.aubakirova@nu.edu.kz (M.A.); nurgul.yegembayeva@nu.edu.kz (N.Y.); 2 Department of Anesthesiology and Intensive Care, National Research Oncology Center, Astana 010000, Kazakhstan; 3 School of Engineering and Digital Sciences, Nazarbayev University, Astana 010000, Kazakhstan; fatima.nabidollayeva@nu.edu.kz (F.N.); yerkin.abdildin@nu.edu.kz (Y.A.); 4 Department of Anesthesia and Intensive Care, University La Sapienza, 00185 Rome, Italy; bilotta@tiscali.it; 5 Department of Anaesthesiology and Intensive Care, Hospital Clìnico Universitario de Valencia, University of Valencia, 46003 Valencia, Spain

**Keywords:** ketamine, neurocognitive dysfunction, delirium, postoperative cognitive dysfunction, anesthesia

## Abstract

Background: Neurocognitive alterations in the perioperative period might be caused by a wide variety of factors including pain, blood loss, hypotension, hypoxia, micro- and macroemboli, cardiopulmonary bypass (CPB), reperfusion damage, and surgery itself, and all are risk factors for developing postoperative delirium (POD) and postoperative cognitive dysfunction (POCD). The objective of this study was to evaluate the effect of ketamine on neurocognitive dysfunction after anesthesia. Methods: We conducted a meta-analysis of randomized controlled trials (RCTs) comparing ketamine use (experimental group) with placebo (controls). Results: The model favors the control group over the experimental group in terms of frequency of hallucinations (the risk ratio with 95% CI is 1.54 [1.09, 2.19], *p*-value = 0.02), the number of patients readmitted within 30 days (RR with 95% CI is 0.25 [0.09, 0.70]), and the number of adverse events (overall RR with 95% CI is 1.31 [1.06, 1.62]). In terms of morphine consumption, the model favors the experimental group. Conclusion: There was no statistically significant difference in incidences of postoperative delirium, vasopressor requirement, and fentanyl consumption between the ketamine and control groups. However, hallucinations were more frequently reported in the ketamine group.

## 1. Introduction

Neurocognitive alterations in the perioperative period might be caused by a wide variety of factors including anesthesia and surgery itself [1]. Many perioperative factors, including pain, blood loss, hypotension, hypoxia, micro- and macroemboli, cardiopulmonary bypass (CPB), reperfusion damage, and surgery itself are risk factors for developing postoperative delirium (POD) and postoperative cognitive dysfunction (POCD) [2]. The incidence of POD varies from 10% to 80% and usually occurs during the first 72 h following surgery. Only 4% of ageing patients who developed POD fully recover at discharge, and up to 80% still have residual impairment at 6 months or later [2]. The incidence of POCD in older patients undergoing cardiac surgeries has been reported to range from 20% to 50% three months after surgery and may reach 55% in those undergoing some other major surgeries [2,3,4].

Management of POD is still empirical. There is some evidence that ketamine might be an option to reduce the risk of POD and POCD in surgical patients. Ketamine is a N-methyl-D-aspartic acid (NMDA) antagonist and has strong anti-nociceptive properties by impacting central sensitization and pain modulation [3,4]. Previous studies have found that ketamine given during subanesthetic intraoperative anesthesia lowers the levels of postoperative inflammation markers, opioid use, and pain. Despite reported hallucinogenic effects that might induce or exacerbate postoperative delirium, ketamine has been recently reported to have protective effects against postoperative neurocognitive dysfunction [3,4,5]. In both human and animal trials, ketamine has demonstrated substantial anti-inflammatory effects and the potential to lessen postoperative delirium [3,4,5]. A recent study showed that ketamine added to common anesthetics might lower POD in cardiac surgery to 3%, as opposed to 31% in the placebo group [6]. Additionally, by reducing the inflammation after surgery simultaneously with the central nervous system, ketamine may provide neuroprotection [4,7]. Contrarily, a large-scale trial in which a single dosage of ketamine was administered during induction during both non-cardiac and cardiac surgery found no difference in the results [6]. Especially when administered alone, the usage of ketamine might be exacerbated by side effects such as disorientation, euphoria, cognitive impairment, and perceptual problems [8].

This systematic review and meta-analysis aimed to compare the effect of ketamine and other anesthesia methods on neurocognitive dysfunction after anesthesia.

## 2. Materials and Methods

### 2.1. Protocol

This systematic review and meta-analysis was planned, performed, and reported according to the “Preferred Reporting Items for Systematic Reviews and Meta-Analyses (PRISMA)” guidelines [9]. The protocol was registered in Open Science Framework. We searched for randomized controlled trials (RCTs) published in English, which studied the effect of ketamine on neurocognitive dysfunction after surgery.

**Figure 1:**
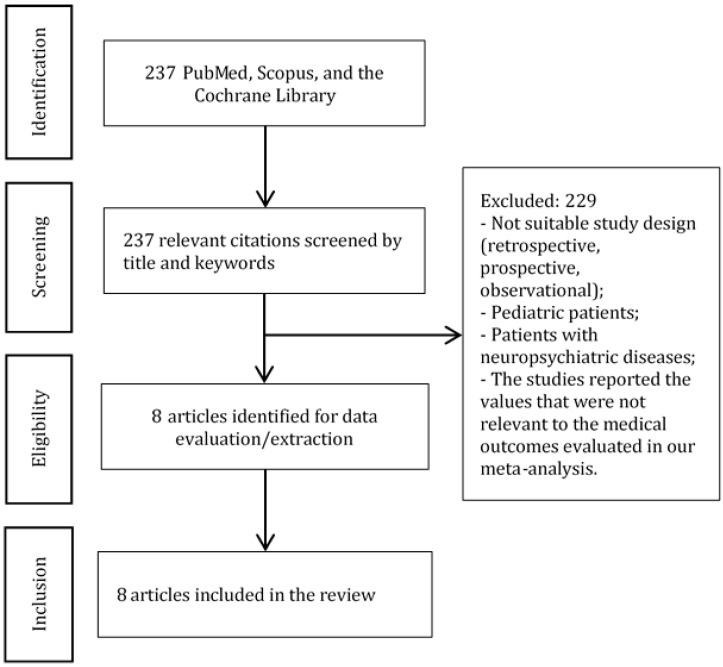
PRISMA diagram. The diagram shows the study selection process.

We searched for relevant articles in PubMed, Scopus, and the Cochrane Library published before March 2023 (Figure 1). The following search terms or their combinations were used during the search: ((((ketamine) AND (anesthesia)) AND (surgery)) AND (postoperative delirium)) AND (postoperative cognitive dysfunction); (“esketamine” OR “esketamine” OR “ketamine” OR “ketamine” OR “ketamin” OR “ketamine s” OR “ketamines”) AND (“anaesthesia” OR “anesthesia” OR “anesthesia” OR “anaesthesias” OR “anesthesias”) AND (“surgery” OR “surgery” OR “surgical procedures, operative” OR (“surgical” AND “procedures” AND “operative”) OR “operative surgical procedures” OR “general surgery” OR (“general” AND “surgery”) OR “general surgery” OR “surgery” OR “surgerys” OR “surgeries”) AND ((“postoperative period” OR (“postoperative” AND “period”) OR “postoperative period” OR “postop” OR “postoperative” OR “postoperatively” OR “postoperatives”) AND (“delirium” OR “delirium” OR “deliriums”)) AND (“postoperative cognitive complications” OR (“postoperative” AND “cognitive” AND “complications”) OR “postoperative cognitive complications” OR (“postoperative” AND “cognitive” AND “dysfunction”) OR “postoperative cognitive dysfunction”).

### 2.2. Participants and Population

#### Inclusion criteria:

- Study types: RCTs;
- Study arms: comparison of ketamine and placebo;
- Age of patients: 18 years and older;
- Surgery: any type of surgery;
- Articles published in English.

### 2.3. Outcomes

The primary outcomes of our meta-analysis were the effect of ketamine on postoperative delirium and hallucinations. The secondary outcomes included hemodynamic stability, pain scores, total dose of opioids, and side effects.

### 2.4. Data Extraction and Statistical Methods

Data were extracted using a standardized Excel form. Study characteristics, such as first author, country, year of publication, patient population, intervention, study design, sample size, and outcomes, were extracted into Table 1. 

### 2.5. Assessment of Methodological Quality

First, the methodological quality of the included studies was assessed using the Oxford quality scoring system (Jadad Scale). The quality of the studies was graded within “the range from 1 (min) to 5 (max) as low (<3), acceptable (3), good (4), and excellent (5)” [12]. Then, each study was evaluated using the Cochrane risk of bias tool as “high risk”, “some concerns”, or “low risk” [13]. Finally, each outcome was examined for the certainty of evidence using GRADE as “high”, “moderate”, “low”, or “very low” [14].

## 3. Results

We have found 237 citations that matched our search criteria (Figure 1). Eight articles [1,2,3,4,5,7,10,11] with 896 patients were selected for the meta-analysis (Table 1 and Table 2).

Data analysis was conducted using the “Review Manager (RevMan) [Computer program]. Version 5.4. The Cochrane Collaboration, 2020”. Heterogeneity was estimated by the I^2^ statistic. Whenever needed, we used mathematical methods for estimating the sample mean and standard deviation [15,16].

### 3.1. Outcomes

#### 3.1.1. Incidence of Delirium 

**Figure 2:**
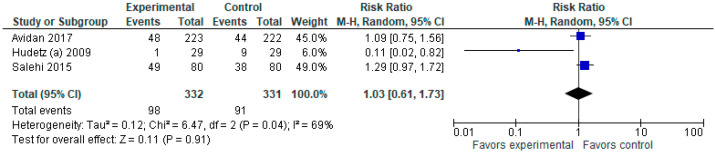
Incidence of delirium. The forest plot shows the pooled risk ratio of the incidence of delirium after ketamine versus placebo use [2,5,10].

There is no statistically significant difference in the frequency of delirium between the experimental (ketamine) and control (placebo) groups (Figure 2).

#### 3.1.2. Incidence of Hallucinations 

**Figure 3:**
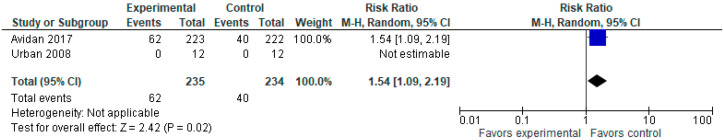
Incidence of hallucinations. The forest plot shows the pooled risk ratio of the incidence of hallucinations after ketamine versus placebo use [5,11].

The model favors the control group over the experimental group in terms of the frequency of hallucinations (Figure 3). However, the result is sensitive to the exclusion of a study by Avidan et al., 2017 [5] in which case there will be no difference between the groups.

#### 3.1.3. Vasopressor Use 

**Figure 4:**
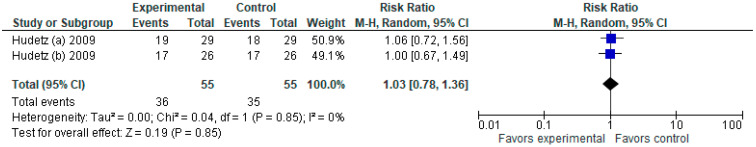
Vasopressor use. The forest plot shows the pooled risk ratio of the incidence of vasopressor use after ketamine versus placebo use [2,4].

The model does not favor either group in terms of the number of patients requiring vasopressors (Figure 4).

#### 3.1.4. Fentanyl Consumption (μg) 

**Figure 5:**
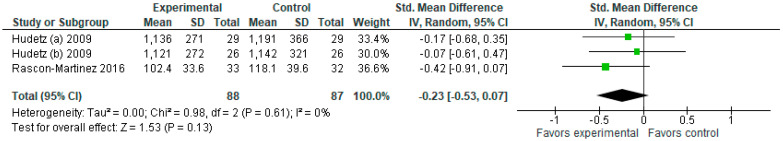
Fentanyl consumption (μg). The forest plot shows the standardized mean difference in fentanyl consumption between the ketamine and the control groups [1,2,4].

The model does not favor either group in terms of fentanyl consumption (μg) at *p* = 0.13 (Figure 5). We should note that Urban et al., 2008 [11] reported data values in mcg/kg, intraoperatively, so we have not included this study here.

#### 3.1.5. Morphine Consumption (mg) 

**Figure 6:**
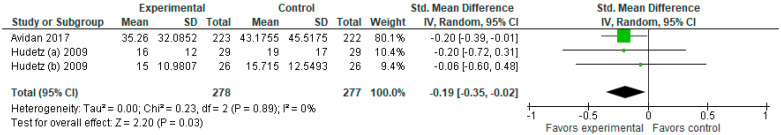
Morphine consumption (mg). The forest plot shows the standardized mean difference in morphine consumption between the ketamine and the control groups [2,4,5].

In terms of morphine consumption (Figure 6), the model favors the experimental group (SMD with 95% CI is −0.19 [−0.35, −0.02]).

#### 3.1.6. Adverse Events 

**Figure 7:**
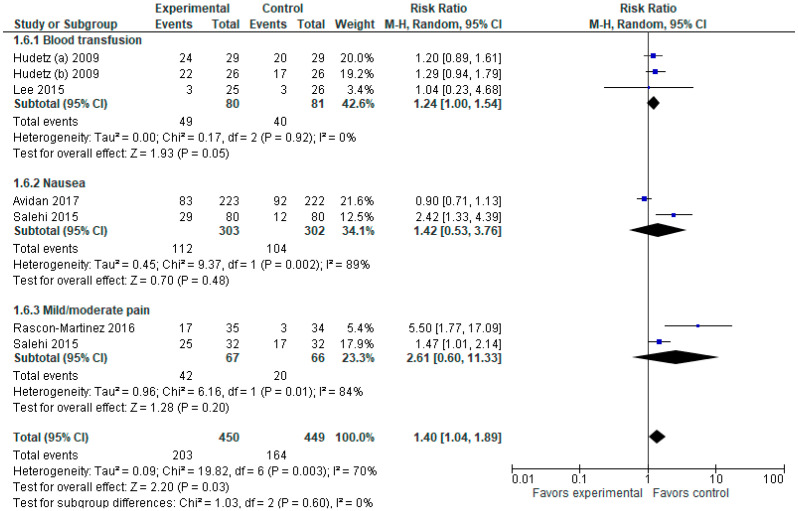
Adverse events. The forest plot shows the pooled risk ratio of the incidence of adverse events (blood transfusion, nausea, mild/moderate pain) after ketamine versus placebo use [1,2,4,5,7,10].

The model tends to favor the control group over the ketamine group. As shown in the forest plot (Figure 7), the overall risk ratio with a 95% CI is 1.31 [1.06, 1.62]. However, the model does not strongly favor the control group over the experimental group in any of the subgroups. The sensitivity analysis shows that the result in incidences of nausea is sensitive to the exclusion of a study by Avidan et al. [5], while the result in pain outcomes is sensitive to the exclusion of Rascon-Martinez et al. [1] and Salehi et al. [10].

#### 3.1.7. Surgery Duration (min) 

**Figure 8:**
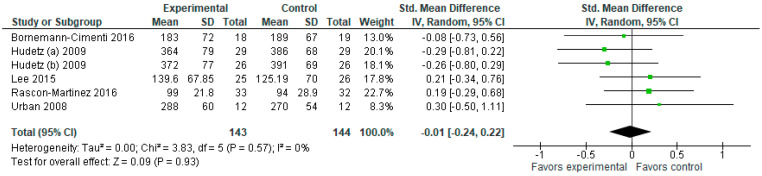
Surgery duration (min). The forest plot shows the standardized mean difference in surgery duration between the ketamine and the control groups [1,2,3,4,7,11].

The model does not favor the ketamine group over the control group in terms of surgery time (Figure 8).

#### 3.1.8. Readmission within 30 Days 

**Figure 9:**
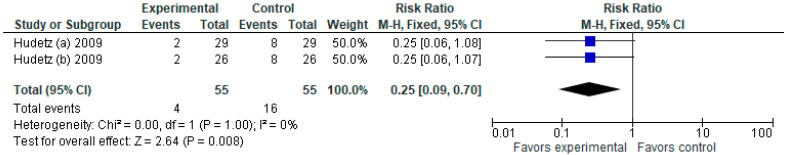
Readmission within 30 days. The forest plot shows the pooled risk ratio of readmission within 30 days after ketamine versus placebo use [2,4].

The model (Figure 9) favors the ketamine group over the control group in terms of the number of patients readmitted within 30 days, RR with a 95% CI is 0.25 [0.09, 0.70].

### 3.2. Assessment of Methodological Quality

As evident from Table 3, of the eight studies, five were rated as “excellent”, two as “good”, and one as “acceptable” quality based on the Oxford quality scoring system (Jadad Scale). Based on the Cochrane Risk of Bias (Table 4), all the included studies had a “low risk” of bias. Based on GRADE (Table 5), the certainty of evidence ranged from “low” to “high”. Appendix A provides the Evidence profile of the studied outcomes.

## 4. Discussion

Although we found no difference between ketamine and control in the incidence of postoperative delirium, the incidence of hallucinations was higher in the ketamine group. We must note that the result is sensitive to the exclusion of the study by Avidan et al. [5], in which case there will be no difference between the groups.

Since postoperative neurocognitive disorders can be induced by numerous factors, many secondary outcomes of this meta-analysis might be important. Despite ketamine is known to provide more stable hemodynamics compared to other intravenous anesthetics, there were no differences in terms of the number of patients requiring vasopressors. Interestingly, there is also a trend to favor control (not statistically significant) in terms of requirement in blood transfusion. We suppose that patients with blood loss were administered ketamine to maintain more stable hemodynamics. There was no difference between the ketamine and control groups in postoperative pain intensity and nausea. Another important finding was that there was a lower readmission rate in the ketamine group.

The conflicting results might be due to the failure of large studies to replicate the results of small studies [5]. Although meta-analyses of small studies might also contradict the findings of large trials, the results of the current meta-analysis support the findings of the large trials. More importantly, POD and POCD are multifactorial conditions, which might be caused by the following factors [17,18]:

(1) Preoperative (advanced age, neurocognitive deterioration, depression, use of anti-depressants);
(2) Intraoperative (blood loss, hypotension, pain, hypernatremia, hyponatremia, deep anesthesia (if the doses of anesthesia are higher than required, prolonged duration of anesthesia and surgery);
(3) Postoperative factors (sleep deprivation, severe pain, electrolyte dysbalance, cerebrovascular events).

Therefore, it is challenging to consider all these factors during the study and patient enrollment. 

It is also important to mention that ketamine has several effects, such as anesthetic, analgesic, antidepressant, anti-inflammatory, and neuroprotective [5,19,20,21]. Ketamine is known for inducing general (dissociative) anesthesia. Ketamine can be also used for local anesthesia as an adjunct to local anesthetics. Dissociative anesthesia is a type of anesthesia lacking complete unconsciousness and characterized by catalepsy, catatonia, and amnesia. Adequate analgesia can be achieved by using low subanesthetic doses (0.15–0.25 mg/kg) for the reduction of acute and chronic pain. Ketamine administration in pre- or intraoperative periods has been used to improve postoperative outcomes due to its ability to reduce the excessive production of proinflammatory cytokines, such as tumor necrosis factor-α, nuclear factor-kB, C-reactive protein, interleukin 6 (IL-6), and inducible nitric oxide synthase. Moreover, the anti-inflammatory effects of ketamine were attributed not only to local but also to systemic anti-inflammatory action [21]. Ketamine also has a preconditioning effect via the inhibition of NMDA receptors, and it was also shown to reduce post-ischemic cortical neuronal loss associated with glutamate-induced calcium overload [7]. Some previous studies also showed that the NMDA receptor through anti-inflammatory mechanisms plays a direct role in the recognition and short-term memory; however, other reports assign this effect to improved cerebral blood flow [22,23,24]. This binding of ketamine to the NMDA receptor suppresses the expression of the factor kβ2 that is involved in the transcription of genes that affects proinflammatory cytokines, including interleukins 6 and 8, and tumor necrosis factor α leading to reduced neuronal apoptosis. Subsequently, ketamine also activates the sympathetic nervous system leading to increased cerebral perfusion pressure [25,26]. Since elevated levels of some cytokines, such as IL-6, have been associated with poor postoperative outcomes, this effect of ketamine seems promising, but more RCTs are required. Ketamine has been reported to modulate abnormal inflammatory substances in major depressive disorder. Ketamine acts as an immunomodulatory but not as an immunosuppressive agent, which might be of particular importance since ketamine is usually used during the induction of anesthesia. 

Although ketamine might have promising positive effects, it also has several psychotomimetic and dissociative adverse effects. Ketamine broadly influences consciousness and perception. Some patients report dissociative and extracorporeal experiences and illusions (being out-of-body) [21]. The most common psychoactive effects are dissociative effects (visual, auditory, and somatosensory stimuli), positive psychotomimetic (hallucinations, conceptual disorganization, unusual thought content, suspiciousness), as well as negative psychotomimetic (emotional withdrawal, motor retardation, and blunted affect). Memory and cognitive impairment effects of ketamine were also reported [5,19,20,21]. 

This meta-analysis has several important limitations. The first limitation of this meta-analysis was the inclusion of the original studies with small sample sizes. The second limitation was heterogeneity in reporting rubrics; therefore, we could not incorporate the results of these studies in the forest plots. The next limitation was no proper assessment or reporting of the side effects. For example, the side effects were observed within 24 h after surgery. However, some side effects might occur later, but they were not assessed or reported. 

## 5. Conclusions

The incidence of postoperative delirium did not differ significantly between the ketamine and placebo groups. However, hallucinations were more frequently reported in the ketamine group. There was no statistically significant difference in fentanyl consumption and vasopressor requirement between the ketamine and control groups. Due to the limitations of the existing trials, future large RCTs are warranted to establish more convincing evidence regarding the protective effects of ketamine against neurocognitive dysfunction. 

## Figures and Tables

**Table 1:** Characteristics of the included studies. Abbreviations: CABG, coronary artery bypass graft; IV, intravenous(ly).

First Author Year	Study Goals	Procedure	Ketamine Dose	Conclusions
Avidan 2017 [5]	To assess the effect of ketamine on delirium in the post-surgical elderly	Various types of surgeries	Low-dose: 0.5 mg/kg High-dose: 1.0 mg/kg	No lowering effect of ketamine on delirium, opioid use, or pain
Bornemann-Cimenti 2016 [3]	To evaluate the use of low-dose ketamine as part of multimodal analgesia to reduce pain and opioid consumption	Elective colorectal and hepatic surgery	Low-dose: 0.25 mg/kg bolus and 0.125 mg/kg/h infusion for 48 h Minimal-dose: 0.015 mg/kg/h infusion following a saline bolus	Minimal dose ketamine had no superiority on pain control or opioid consumption, but it lowered the incidence of delirium
Hudetz 2009 [2]	To examine the effect of ketamine on the incidence of delirium in older cardiac surgery patients	CABG, valvular surgery, coronary artery disease, valvular disease	0.5 mg/kg IV	Incidence of delirium was lower in the ketamine group
Hudetz 2009 [4]	To assess the incidence of delirium following ketamine use in cardiac surgery	Cardiac surgery	0.5 mg/kg IV	Ketamine reduced post-operative cognitive dysfunction seven days after cardiac surgery
Lee 2015 [7]	To assess the effect of ketamine on postoperative cognitive dysfunction in orthopedic surgery patients	Acromioplasty, open reduction and internal fixation, spine surgery, total hip replacement arthroplasty, total knee replacement arthroplasty/Carpal tunnel syndrome, fractures	Ketamine: 0.5 mg/kg IV Placebo: 3 mL 0.9% saline	No effect of ketamine on postoperative cognitive dysfunction
Rascon-Martinez 2016 [1]	To examine the effect of ketamine on cognitive status in the elderly	Ophthalmic surgery: vitrectomy or cataract surgery/Cataract	0.3 mg/kg dose	Improved cognitive status following ketamine use
Salehi 2015 [10]	To study the effect of ketamine on delirium and depression after electro-convulsive therapy	Electroconvulsive therapy/Drug-resistant major depression	Ketamine 0.8 mg/kgSodium thiopental 1–1.5 mg/kg	Comparable effect on depression, higher incidence of complications in the ketamine group
Urban 2008 [11]	To study the effect of ketamine as part of multimodal analgesia for managing acute pain following spinal fusions	Spinal fusions	0.2 mg/kg on induction; 2 mcg/kg/hour for the next 24 h	Improved pain scores in the ketamine group, but no difference in opioid consumption or cognitive function

**Table 2:** Abbreviations: RCT, randomized controlled trial.

First Author Year	Study Design	Country	Groups	Age	Sample Size
Avidan 2017 [5]	RCT	USA, Korea, Canada, India	Placebo Low-dose ketamine High-dose ketamine	70 (6.9) 70 (7.2) 70 (7.3)	654 (217/221/216)
Bornemann-Cimenti 2016 [3]	RCT	Austria	Low-dose Minimal dose Placebo	62.2 (9.8) 58.4 (8.1) 61 (12.4)	56 (19/18/19)
Hudetz 2009 [2]	RCT	USA	Placebo Ketamine	60 (8) 68 (8)	58 (29/29)
Hudetz 2009 [4]	RCT	USA	Placebo Ketamine Control nonsurgical	67 (8) 68 (7) 64 (7)	78 (26/26/26)
Lee 2015 [7]	RCT	Korea	Ketamine Placebo	68.38 (6.54) 68.32 (5.34)	51 (25/26)
Rascon-Martinez 2016 [1]	RCT	Mexico	Ketamine Placebo	70.5 (4.7) 68.7 (7.1)	65 (33/32)
Salehi 2015 [10]	RCT	Iran	Ketamine Sodium thiopental	20–60	160 (80/80)
Urban 2008 [11]	RCT	USA	Ketamine Control	53 (12) 48 (9)	24 (12/12)

**Table 3:** Methodological quality of the studies (Jadad scale).

Study or Subgroup	Was This Study Described as Randomized?	Was the Method Used to Generate the Sequence of Randomization Appropriate and Described?	Was the Study Described as Double-Blind?	Was the Method of Double Blind Appropriate and Described?	Was there a Description of Withdraw and Dropouts?	Total Score
Avidan 2017 [5]	1	1	1	1	1	5
Bornemann-Cimenti 2016 [3]	1	1	1	1	1	5
Hudetz 2009 [2]	1	1	1	1	0	4
Hudetz 2009 [4]	1	1	1	1	1	5
Lee 2015 [7]	1	1	1	1	1	5
Rascon-Martinez 2016 [1]	1	1	1	0	1	4
Salehi 2015 [10]	1	1	1	0	0	3
Urban 2008 [11]	1	1	1	1	1	5

**Table 4:** Cochrane risk of bias.

Study (First Author, Year)	Risk of Bias Arising from the Randomization Process	Risk of Bias Due to Deviations from the Intended Interventions	Missing Outcome Data	Risk of Bias in Measurement of the Outcome	Risk of Bias in Selection of the Reported Result	Overall Risk of Bias
Avidan et al., 2017 [5]	Low risk	Low risk	Some concerns	Low risk	Low risk	Low risk
Rascón-Martínez et al., 2016 [1]	Low risk	Low risk	Some concerns	Low Risk	Low risk	Low risk
Hudetz et al., 2009 [2]	Low risk	Low risk	Low risk	Low risk	Low risk	Low risk
Hudetz et al., 2009 [4]	Low risk	Low risk	Low risk	Low risk	Low risk	Low risk
Salehi et al., 2015 [10]	Low risk	Low risk	Low risk	Low risk	Low risk	Low risk
Urban et al., 2008 [11]	Low risk	Low risk	Low risk	Low risk	Low risk	Low risk
Lee et al., 2015 [7]	Low risk	Low risk	Some concerns	Low risk	Low risk	Low risk
Bornemann-Cimenti et al., 2016 [3]	Low risk	Low risk	Some concerns	Low risk	Low risk	Low risk

**Table 5:** Summary of findings.

Outcomes	Risk Ratio [95% CI]	Standardized Mean Difference [95% CI]	Number of Participants (Studies)	Certainty of the Evidence (GRADE)
Incidence of delirium	1.03 [0.61, 1.73]	-	663 (3)	Low ⨁⨁⊖⊖
Incidence of hallucinations	1.54 [1.09, 2.19]	-	469 (2)	High ⨁⨁⨁⨁
Morphine consumption (mg)	-	−0.19 [−0.43, 0.06]	377 (3)	High ⨁⨁⨁⨁
Overall adverse events	1.40 [1.04, 1.89]	-	899 (6)	High ⨁⨁⨁⨁
Fentanyl consumption (μg)	-	−0.23 [−0.53, 0.07]	175 (3)	Low ⨁⨁⊖⊖
Vasopressor use	1.03 [0.78, 1.36]	-	110 (2)	Moderate ⨁⨁⨁⊖
Readmissions within 30 days	0.25 [0.09, 0.70]	-	110 (2)	Moderate ⨁⨁⨁⊖

Symbols: ⊕⊕⊕⊕—“high certainty”, ⊕⊕⊕⊖—“moderate certainty” and ⊕⊕⊖⊖—“low certainty”.

## Data Availability

The data presented in this study are available on request from the corresponding author.

## Appendix A

**Table A1:** Evidence profile.

	Incidences of Delirium	Incidences of Hallucinations	Vasopressor Use	Fentanyl Consumption (μg)	Morphine Consumption (mg)	Overall Adverse Events	Readmissions within 30 Days
Risk of bias	Not serious	Not serious	Not serious	Serious	Not serious	Not serious	Not serious
Lack of allocation concealment	No	No	No	No	No	No	No
Lack of blinding	No	No	No	No	No	No	No
Incomplete accounting of patients and outcome events	No	No	No	Yes	No	No	No
Selective outcome reporting	No	No	No	No	No	No	No
Other limitations	No	No	No	No	No	No	No
Inconsistency	Very serious	Serious	Not serious	Not serious	Not serious	Not serious	Not serious
I^2^ (unexplained heterogeneity of results)	Yes	No	No	No	No	No	No
Wide variance of point estimates	Yes	No	No	No	No	Yes	No
Confidence intervals (CIs) do not overlap	Yes	No	No	No	No	No	No
Indirectness	Not serious	Not serious	Not serious	Not serious	Not serious	Not serious	Not serious
Differences in population	No	No	No	No	No	No	No
Differences in interventions	No	No	No	No	No	No	No
Differences in outcome measures	No	No	No	No	No	No	No
Indirect comparisons	No	No	No	No	No	No	No
Imprecision	Serious	Serious	Very serious	Serious	Not serious	Not serious	Serious
Few patients	No	No	Yes	Yes	No	No	Yes
Wide confidence interval (CI)	Yes	No	Yes	No	No	No	No
Other considerations	None	None	None	None	None	None	Not serious
RR > 2 or RR < 0.5 RR > 5 or RR < 0.2	No	No	No	No	No	No	No
Dose-response gradient	No	No	No	No	No	No	No
Effect of plausible residual confounding	No	No	No	No	No	No	No

## References

[1] Rascón-Martínez D.M., Fresán-Orellana A., Ocharán-Hernández M.E., Genis-Zarate J.H., Castellanos-Olivares A. (2016). The Effects of Ketamine on Cognitive Function in Elderly Patients Undergoing Ophthalmic Surgery: A Pilot Study. Anesth. Analg..

[2] Hudetz J.A., Patterson K.M., Iqbal Z., Gandhi S.D., Byrne A.J., Hudetz A.G., Warltier D.C., Pagel P.S. (2009). Ketamine Attenuates Delirium after Cardiac Surgery with Cardiopulmonary Bypass. J. Cardiothorac. Vasc. Anesth..

[3] Bornemann-Cimenti H., Wejbora M., Michaeli K., Edler A., Sandner-Kiesling A. (2016). The Effects of Minimal-Dose versus Low-Dose S-Ketamine on Opioid Consumption, Hyperalgesia, and Postoperative Delirium: A Triple-Blinded, Randomized, Active- and Placebo-Controlled Clinical Trial. Minerva Anestesiol..

[4] Hudetz J.A., Iqbal Z., Gandhi S.D., Patterson K.M., Byrne A.J., Hudetz A.G., Pagel P.S., Warltier D.C. (2009). Ketamine Attenuates Post-Operative Cognitive Dysfunction after Cardiac Surgery. Acta Anaesthesiol. Scand..

[5] Avidan M.S., Maybrier H.R., Abdallah A.B., Jacobsohn E., Vlisides P.E., Pryor K.O., Veselis R.A., Grocott H.P., Emmert D.A., Rogers E.M. (2017). Intraoperative Ketamine for Prevention of Postoperative Delirium or Pain after Major Surgery in Older Adults: An International, Multicentre, Double-Blind, Randomised Clinical Trial. Lancet.

[6] Siripoonyothai S., Sindhvananda W. (2021). Comparison of Postoperative Delirium within 24 Hours between Ketamine and Propofol Infusion during Cardiopulmonary Bypass Machine: A Randomized Controlled Trial. Ann. Card. Anaesth..

[7] Lee K.H., Kim J.Y., Kim J.W., Park J.S., Lee K.W., Jeon S.Y. (2015). Influence of Ketamine on Early Postoperative Cognitive Function After Orthopedic Surgery in Elderly Patients. Anesthesiol. Pain Med..

[8] Loo C.K., Katalinic N., Garfield J.B.B., Sainsbury K., Hadzi-Pavlovic D., Mac-Pherson R. (2012). Neuropsychological and Mood Effects of Ketamine in Electroconvulsive Therapy: A Randomised Controlled Trial. J. Affect. Disord..

[9] Page M.J., McKenzie J.E., Bossuyt P.M., Boutron I., Hoffmann T.C., Mulrow C.D., Shamseer L., Tetzlaff J.M., Akl E.A., Brennan S.E. (2021). The PRISMA 2020 Statement: An Updated Guideline for Reporting Systematic Reviews. BMJ.

[10] Salehi B., Mohammadbeigi A., Kamali A., Taheri-Nejad M., Moshiri I. (2015). Impact Comparison of Ketamine and Sodium Thiopental on Anesthesia during Electroconvulsive Therapy in Major Depression Patients with Drug-Resistant; a Double-Blind Randomized Clinical Trial. Ann. Card. Anaesth..

[11] Urban M.K., Ya Deau J.T., Wukovits B., Lipnitsky J.Y. (2008). Ketamine as an Adjunct to Postoperative Pain Management in Opioid Tolerant Patients after Spinal Fusions: A Prospective Randomized Trial. HSS J. ^®^ Musculoskelet. J. Hosp. Spec. Surg..

[12] Jadad A.R., Moore R.A., Carroll D., Jenkinson C., Reynolds D.J., Gavaghan D.J., McQuay H.J. (1996). Assessing the Quality of Reports of Randomized Clinical Trials: Is Blinding Necessary?. Control. Clin. Trials.

[13] Sterne J.A.C., Savović J., Page M.J., Elbers R.G., Blencowe N.S., Boutron I., Cates C.J., Cheng H.-Y., Corbett M.S., Eldridge S.M. (2019). RoB 2: A Revised Tool for Assessing Risk of Bias in Randomised Trials. BMJ.

[14] Guyatt G.H., Oxman A.D., Schünemann H.J., Tugwell P., Knottnerus A. (2011). GRADE Guidelines: A New Series of Articles in the Journal of Clinical Epidemiology. J. Clin. Epidemiol..

[15] Luo D., Wan X., Liu J., Tong T. (2018). Optimally Estimating the Sample Mean from the Sample Size, Median, Mid-Range, and/or Mid-Quartile Range. Stat. Methods Med. Res..

[16] Wan X., Wang W., Liu J., Tong T. (2014). Estimating the Sample Mean and Standard Deviation from the Sample Size, Median, Range and/or Interquartile Range. BMC Med. Res. Methodol..

[17] Viderman D., Brotfain E., Bilotta F., Zhumadilov A. (2020). Risk factors and mechanisms of postoperative delirium after intracranial neurosurgical procedures. Asian J. Anesthesiol..

[18] Viderman D., Nabidollayeva F., Aubakirova M., Yessimova D., Badenes R., Abdildin Y. (2023). Postoperative Delirium and Cognitive Dysfunction after General and Regional Anesthesia: A Systematic Review and Meta-Analysis. J. Clin. Med..

[19] Glumac S., Kardum G., Karanovic N. (2019). Postoperative Cognitive Decline After Cardiac Surgery: A Narrative Review of Current Knowledge in 2019. Med. Sci. Monit..

[20] Dale O., Somogyi A.A., Li Y., Sullivan T., Shavit Y. (2012). Does Intraoperative Ketamine Attenuate Inflammatory Reactivity Following Surgery? A Systematic Review and Meta-Analysis. Anesth. Analg..

[21] Zanos P., Moaddel R., Morris P.J., Riggs L.M., Highland J.N., Georgiou P., Pereira E.F.R., Albuquerque E.X., Thomas C.J., Zarate C.A. (2018). Ketamine and Ketamine Metabolite Pharmacology: Insights into Therapeutic Mechanisms. Pharmacol. Rev..

[22] Malhotra A.K., Pinals D.A., Weingartner H., Sirocco K., Missar C.D., Pickar D., Breier A. (1996). NMDA Receptor Function and Human Cognition: The Effects of Ketamine in Healthy Volunteers. Neuropsychopharmacology.

[23] Holcomb H.H., Lahti A.C., Medoff D.R., Weiler M., Tamminga C.A. (2001). Sequential Regional Cerebral Blood Flow Brain Scans Using PET with H2^15^O Demonstrate Ketamine Actions in CNS Dynamically. Neuropsychopharmacology.

[24] Sakai T., Ichiyama T., Whitten C.W., Giesecke A.H., Lipton J.M. (2000). Ketamine Suppresses Endotoxin-Induced NF-KappaB Expression. Can. J. Anaesth. J. Can. Anesth..

[25] Baldwin A.S. (1996). The NF-Kappa B and I Kappa B Proteins: New Discoveries and Insights. Annu. Rev. Immunol..

[26] Långsjö J.W., Kaisti K.K., Aalto S., Hinkka S., Aantaa R., Oikonen V., Sipilä H., Kurki T., Silvanto M., Scheinin H. (2003). Effects of Subanesthetic Doses of Ketamine on Regional Cerebral Blood Flow, Oxygen Consumption, and Blood Volume in Humans. Anesthesiology.

